# The gut microbiome in human health and disease—Where are we and where are we going? A bibliometric analysis

**DOI:** 10.3389/fmicb.2022.1018594

**Published:** 2022-12-15

**Authors:** Zhiqiang Huang, Kun Liu, Wenwen Ma, Dezhi Li, Tianlu Mo, Qing Liu

**Affiliations:** School of Health Science and Engineering, University of Shanghai for Science and Technology, Shanghai, China

**Keywords:** gut microbiota, research trend, hot topics and frontiers, visualized analysis, human health and disease

## Abstract

**Background:**

There are trillions of microbiota in our intestinal tract, and they play a significant role in health and disease *via* interacting with the host in metabolic, immune, neural, and endocrine pathways. Over the past decades, numerous studies have been published in the field of gut microbiome and disease. Although there are narrative reviews of gut microbiome and certain diseases, the whole field is lack of systematic and quantitative analysis. Therefore, we outline research status of the gut microbiome and disease, and present insights into developments and characteristics of this field to provide a holistic grasp and future research directions.

**Methods:**

An advanced search was carried out in the Web of Science Core Collection (WoSCC), basing on the term “gut microbiome” and its synonyms. The current status and developing trends of this scientific domain were evaluated by bibliometric methodology. CiteSpace was used to perform collaboration network analysis, co-citation analysis and citation burst detection.

**Results:**

A total of 29,870 articles and 13,311 reviews were retrieved from the database, which involve 42,900 keywords, 176 countries/regions, 19,065 institutions, 147,225 authors and 4,251 journals. The gut microbiome and disease research is active and has received increasing attention. Co-cited reference analysis revealed the landmark articles in the field. The United States had the largest number of publications and close cooperation with other countries. The current research mainly focuses on gastrointestinal diseases, such as inflammatory bowel disease (IBD), ulcerative colitis (UC) and Crohn’s disease (CD), while extra-intestinal diseases are also rising, such as obesity, diabetes, cardiovascular disease, Alzheimer’s disease, Parkinson’s disease. Omics technologies, fecal microbiota transplantation (FMT) and metabolites linked to mechanism would be more concerned in the future.

**Conclusion:**

The gut microbiome and disease has been a booming field of research, and the trend is expected to continue. Overall, this research field shows a multitude of challenges and great opportunities.

## Introduction

The human gut microbiota originated from colonization by environmental microbes during birth, and live in symbiosis with the host throughout life (Koenig et al., 2011; Kundu et al., 2017). The inoculum source usually and mainly is the mother’s vaginal and fecal microbiomes (Koenig et al., 2011). Human microbiota carried diverse set of genomes, and is considered as human second genome (Grice and Segre, 2012). While the microbes that reside in our gut account for the vast majority, present more than 1,000 species (Almeida et al., 2019), and the number of microorganisms is estimated up to trillions (Sender et al., 2016). These abundant and diverse gut microbes constitute a dynamic and complex ecosystem and perform various functions that are essential for the human host (Heintz-Buschart and Wilmes, 2018). On the one hand, there are competition and cooperation within these microbial consortia (Coyte and Rakoff-Nahoum, 2019), on the other hand, they also interact with the host in multiple aspects, including digestion and metabolism (Krautkramer et al., 2021), immune system (Rooks and Garrett, 2016) and unconscious system (Dinan and Cryan, 2017). Hence, the gut microbiome directly or indirectly impacts the host’s health.

It should be noted that the concept that our resident microbial communities make essential contributions to the host’s physiology and health can date back to Louis Pasteur (1822–1895; Stappenbeck et al., 2002). Indeed, the gut microbiome has been associated with various diseases and conditions in the past decades, such as IBD (Morgan et al., 2012), obesity (Fei and Zhao, 2013), diabetes (Lau et al., 2021), Parkinson’s disease (Wallen et al., 2021) and cancer (Gopalakrishnan et al., 2018). Meanwhile, the gut microbiome shows great promise for disease diagnosis, i.e., as microbial biomarkers with operational taxonomic units (OTUs), taxa and metabolite (Wu et al., 2021); and for disease therapy by manipulation of the gut microbiome, such as dietary interventions, microbial supplements and FMT (Durack and Lynch, 2018).

The role of the gut microbiome in human health and disease has received increasing attention over the last 20 years, and the trend is expected to continue. At present, some fundamental problems need to be addressed in this field. For example, the taxa, genome, functions and cultivation of microbial dark matter (Pasolli et al., 2019; Jiao et al., 2021). Moreover, although many studies have shed light on gut microbiome in health and disease, and established correlations with various diseases in both experimental animals and humans, the causal relationship and molecular mechanisms remain unclear in the most studies. Besides, the application strategies and safety problems in gut microbiome interventions need to be taken into account (Swann et al., 2020). With the biotechnological and computational advancement in this field, more and further explorations will certainly be conducted.

Currently, the volume of scientific literatures about the gut microbiome and disease presents exponential growth. Although there are narrative reviews of gut microbiome and a specific disease, the entire research filed of the gut microbiome and disease is still lack of systematic and quantitative analysis. It is essential to outline this research domain to provide relevant scholars a ready and holistic grasp. Bibliometrics is a multidisciplinary discipline of quantitative analysis of all knowledge carriers by mathematical and statistical methods (Yu et al., 2018). The number and citations of academic publications can reflect the knowledge structure and development features of a scientific domain. Bibliometric analysis is beneficial for identifying and mapping the cumulative scientific knowledge and evolutionary nuances of scientific fields (Donthu et al., 2021). Bibliometrics has been widely used in many other fields, such as economic management, information science, energy and environment (Yu et al., 2020b). Therefore, we profile the research landscape of gut microbiome and disease with bibliometric methodology, to provide historical context and detect hot topics and emerging areas in this field. Furthermore, future evolutionary paths and challenges in this field are discussed.

## Materials and methods

### Data source and search strategy

Data were retrieved by an advanced search from the WoSCC of Clarivate Analytics,^1^ a curated collection of high-quality academic material on the Web of Science™ platform generally used for literature search, journal selection, research evaluation and bibliometric analysis (Li et al., 2018). To avoid bias due to daily updates of the database, document retrieval and export were performed within a single day (May 1, 2022). In order to include as far as possible relevant publications, synonyms for the gut microbiota and disease were included in the search strategy, and the boolean search was set to TS = [(gut* OR intestin* OR gastrointestin* OR gastro-intestin*) AND (microbiota OR microbiome OR flora OR microflora OR bacteria OR microbe* OR microorganism*)] AND TS = (disease*). The time span of publications was set as 1985-01-01 to 2021-12-31. The full record and cited references of the retrieved documents were saved for further analysis. The workflow of the study was presented in Supplementary Figure 1.

### Bibliometric analysis and data visualization

Given that original research is considered as primary literature and presents new knowledge to a certain research area, the “Articles” type of documents was used to evaluate the trends and hotspots of the gut microbiome and disease research. Citespace (Chen et al., 2012; v5.8.R3) was used to analyze reference co-citation, keyword co-occurrence, keywords burst and cooperation relationships among countries, institutions and authors. The Gephi (Bastian et al., 2009; v.0.9.2) was used to construct network graphs.

## Results

### Research trend of gut microbiome in human health and disease

#### The increase of publications number and subject categories

**Figure 1:**
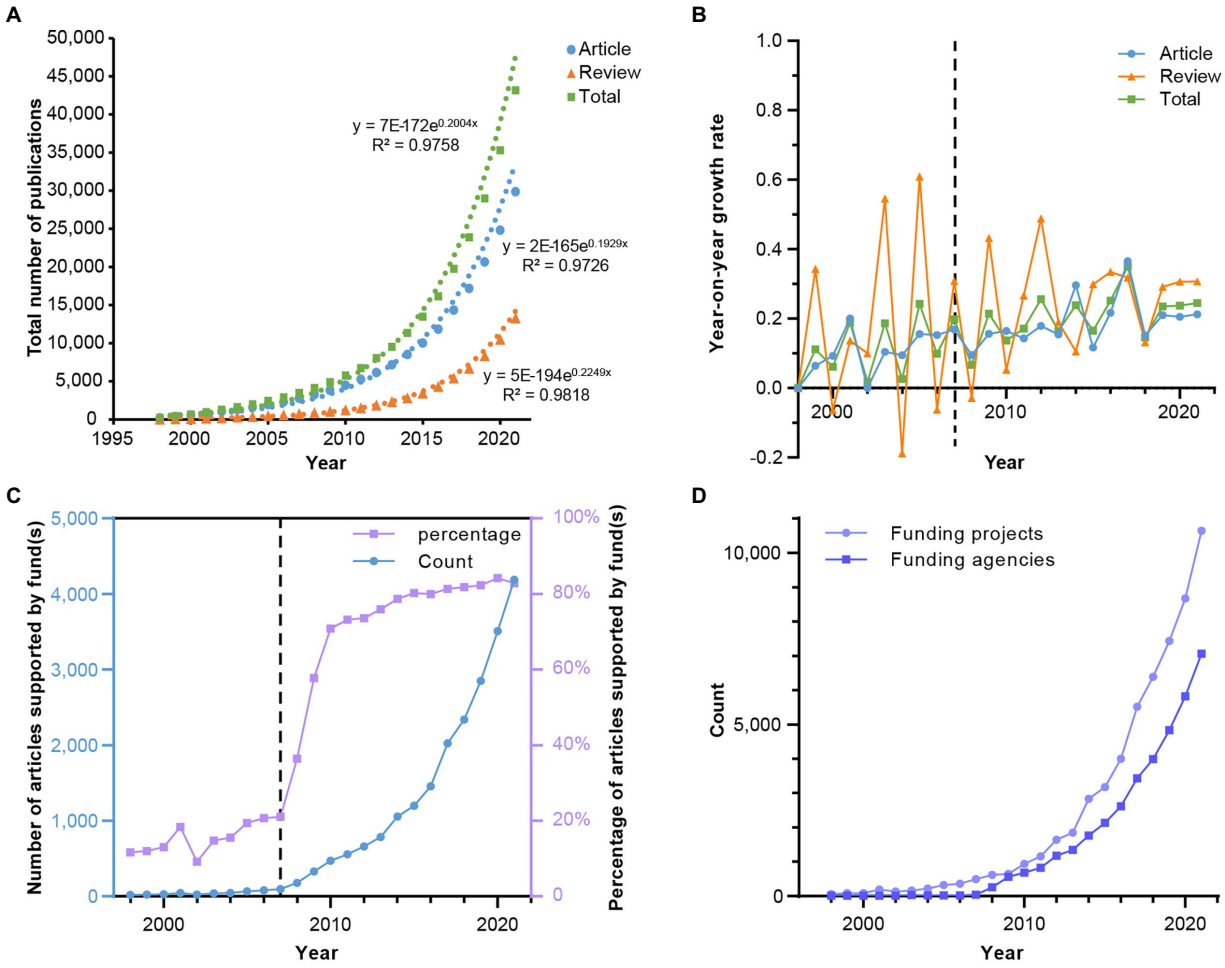
The trend of publications and funding in the research field of gut microbiome and disease. **(A)** The cumulative number of publications in each year and their exponential regressions. **(B)** The year-on-year growth rate of publications. **(C)** The number and percentage of funded articles. **(D)** The number of funding agencies and projects each year.

A total of 45,207 academic publications were retrieved from WoSCC, and publication years were distributed from 1996 to 2021. Among these publications, articles account for 66.074% (29,870 records), reviews account for 29.445% (13,311 records), other document types and their percentages see Supplementary Table 1. The overall output of publications has increased approximately exponentially for the last two decades (Figure 1A). Most of the studies were reported in the recent 15 years (n = 27,558, 92.260%). A turning point can be observed around 2007 (Figures 1B,C), since that, the number of publications has been rising drastically. This is partially because of the invention of next-generation sequencing technologies. Other important reasons are the completion of the Human Genome Project (HGP) the launch of the Human Microbiome Project (HMP) and the Metagenomics of The Human Intestinal Tract (MetaHIT). The number of articles supported by fund(s), funding agencies, and funding projects has also been increasing for 26 years (Figures 1C,D), and the percentage of articles supported by fund(s) has been up to 80% in recent 5 years. These results reveal that the gut microbiome and disease research is active and has received increasing attention.

A variety of web of science categories (174/254) are involved in these published articles (29,870), and the number was gradually ascending to 130 in 2021 (Supplementary Figure 2A), which suggests that the scientific field presents interdisciplinary characteristics (Supplementary Figures 3, 4A–D). The top 10 subject categories are Microbiology, Immunology, Multidisciplinary Sciences, Gastroenterology & Hepatology, Biochemistry & Molecular Biology, Nutrition & Dietetics, Food Science & Technology, Pharmacology & Pharmacy and Biotechnology & Applied Microbiology, and the co-occurrence network of subject categories in the recent 5 years is shown in Supplementary Figure 2B. This research area shows tight relationships with medicine, immunology and nutrition besides microbiology (Supplementary Figures 2C, 5). There is remarkable growth in the number of articles related to cancer and the nerve system every year (Supplementary Figures 2D, 5).

#### The shift of research topics

**Figure 2:**
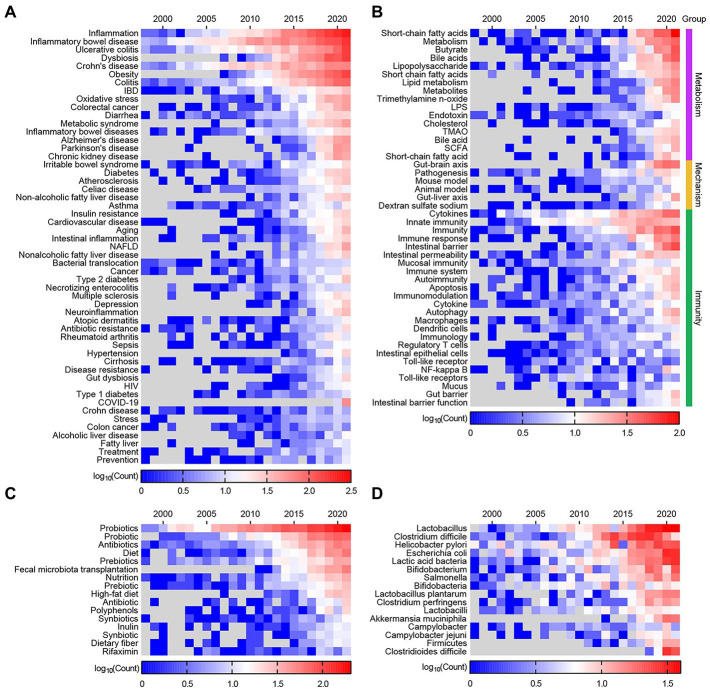
Heatmap of the top 200 keywords by frequency from articles published in 1998–2021. **(A)** The keywords related to “Diseases/Conditions.” **(B)** The keywords related to “Immunity,” “Mechanism,” and “Metabolism.” **(C)** The keywords related to “Intervention.” **(D)** The keywords related to “Microbes.”

The top 200 out of 33,664 keywords by frequency in 1996–2021 were used to construct heatmaps. These keywords were classified into nine categories, including “Definition,” “Technology,” “Experimental subjects” (Supplementary Figure 6), “Diseases/Conditions,” “Immunity,” “Mechanism,” “Metabolism,” “Intervention,” and “Microbes” (Figure 2). Description about this scientific area shifts gradually from “microflora” to “microbiota” and “microbiome” (Supplementary Figure 6A). A technological transition from PCR to sequencing and omics technologies is detected (Supplementary Figure 6B). The primary research subjects include “child,” “infant,” “pregnancy,” and “mice” (Supplementary Figure 6C).

Intestinal diseases have higher keywords frequency than others (Figure 2A). Additionally, most intestinal diseases cover almost the whole period in this research area, and part of them remain hot topics with high keywords frequency, such as “Inflammatory bowel disease,” “ulcerative colitis,” and “Crohn’s disease.” This is easy to understand, considering that the intestines provide a natural habitat for these microorganisms and exchange substances with them. While extra-intestinal diseases draw scientists’ attention in the later years, such as obesity, diabetes, Alzheimer’s disease, Parkinson’s disease, cardiovascular disease, hypertension and depression. Due to the COVID-19 pandemic, the connection between it and the gut microbiota was also established (Figure 2A). Metabolism-related topics with the highest focus are short chain fatty acids (SCFA), butyrate, bile acid and trimethylamine N-oxide (TMAO). Hot topics related to immunity are cytokines, innate immunity and intestinal barrier (Figure 2B). Probiotics, antibiotics, diet and prebiotics are popular topics in invention of gut microbiome, while FMT and high fat diet are emerging topic (Figure 2C). In this field, the primary concern of microbes are probiotics and intestinal pathogens (Figure 2D). Supplementary Figure 7 shows the changing trend in the top 15 keywords over time.

**Figure 3:**
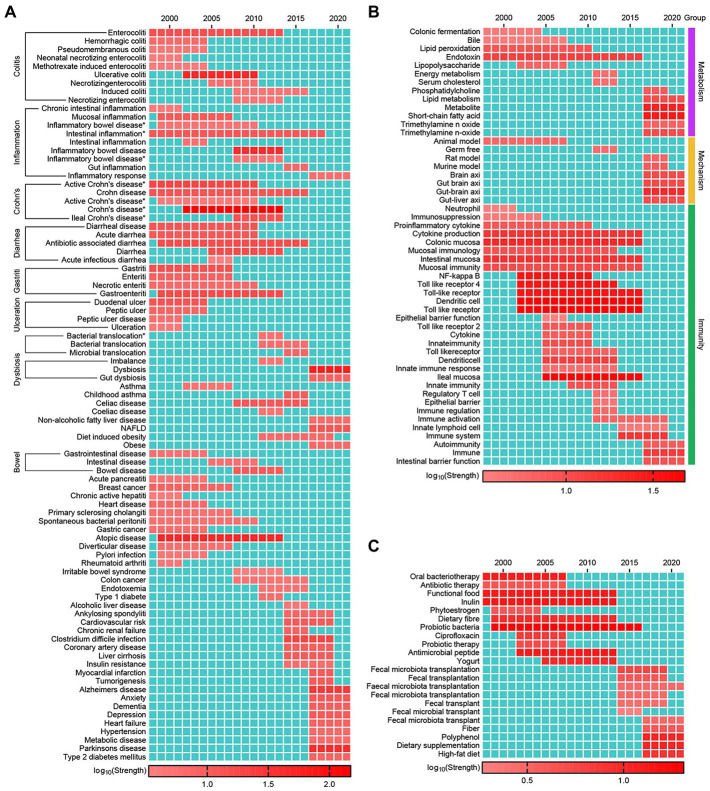
Keywords with strongest bursts from 1998 to 2021. **(A)** The keywords related to “Diseases/Conditions.” **(B)** The keywords related to “Immunity,” “Mechanism,” and “Metabolism.” **(C)** The keywords related to “Intervention.” Asterisk (*) indicate the origin words missed single quotation marks or blank and has been corrected. The red bars indicate burst duration and strength.

Keywords burst means the sudden increase of keywords frequency in a specific period, which involves two attributes—burst strength and duration. A total of 726 keywords were detected as burst keywords. These keywords were also classified into seven categories, i.e., “Definition,” “Technology” (Supplementary Figure 8), “Diseases/Conditions,” “Metabolism,” “Immunity,” “Mechanism,” and “Intervention” (Figure 3). Description and technological shift in the development of the field are also observed (Supplementary Figure 8). 16S rRNA sequencing has become the most useful and active technique to decipher the diversity and abundance of the microbiome.

The top 5 burst keywords related to disease with the highest burst strength are “Crohn’s disease,” “dysbiosis,” “atopic disease,” “ulcerative colitis,” and “Parkinson’s disease.” “Intestinal inflammation” has the longest burst duration (1998–2018) followed by “Crohn’s disease” and “diarrhea.” Overall burst keywords related to intestinal disease covered the early and middle period (−2013) such as “enterocolitis,” “Crohn’s disease,” and “diarrhea”; while extra-intestinal diseases take up the later period (2014–2021) such as “obesity,” “cardiovascular disease,” “Alzheimer’s disease,” “anxiety,” “Parkinson’s disease,” “dementia,” “depression,” “hypertension,” and “type 2 diabetes mellitus” (Figure 3A). The burst keywords involving “Immunity,” “Mechanism,” and “Metabolism” are presented in Figure 3B. Among them “colonic fermentation,” “bile,” and “lipopolysaccharide” burst at early period. On the contrary “SCFA,” “TMAO,” and “phosphatidylcholine” are detected as burst keywords in recent years. As for the intervention of the gut microbiome “FMT,” “fiber,” “dietary supplementation” and high-fat diet are identified as burst keywords over the last several years (Figure 3C).

#### Knowledge map of gut microbiome and disease

**Figure 4:**
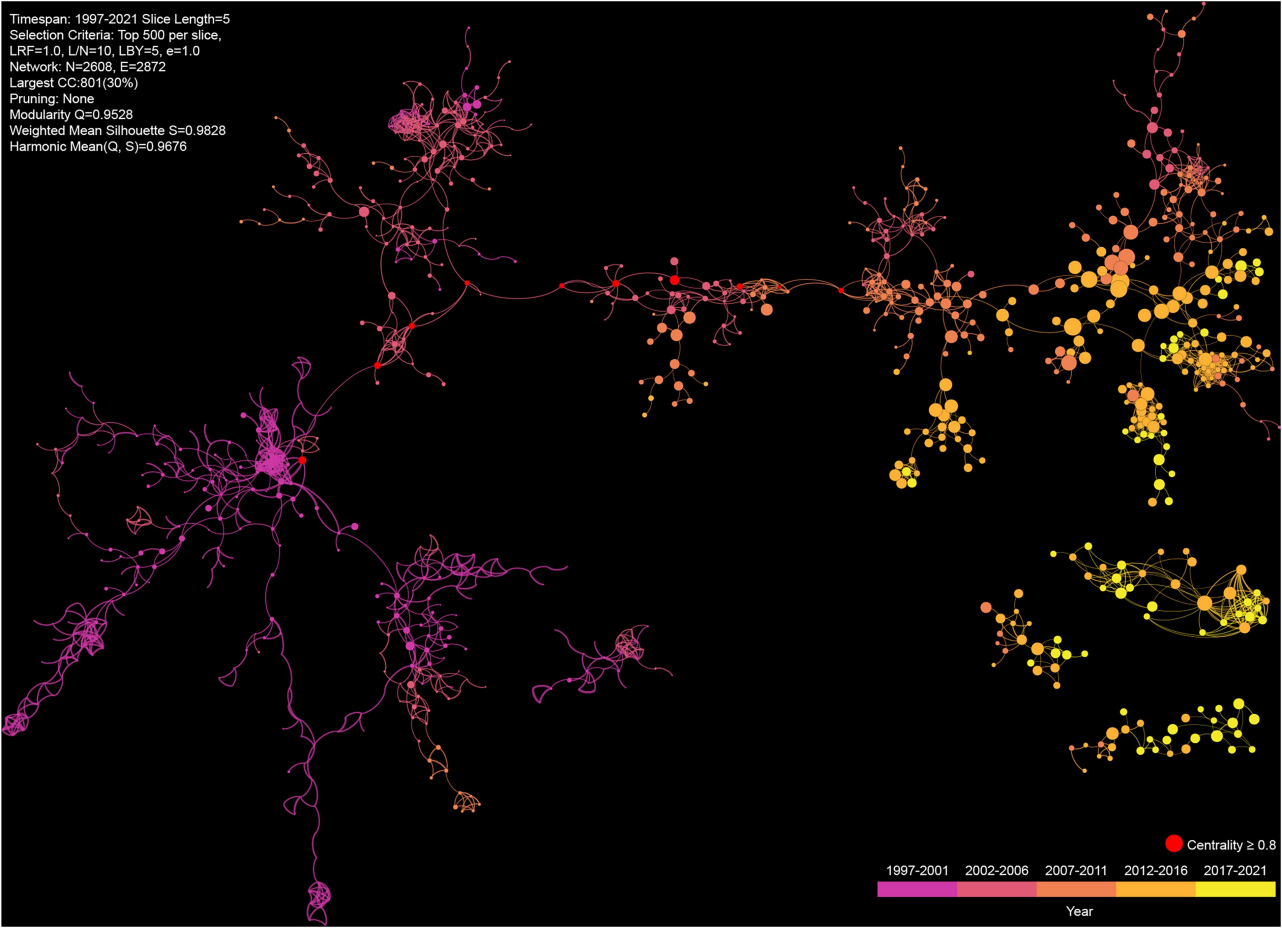
The top 5 largest components of co-citation network on the gut microbiome and disease between 1997 and 2021. Each node represents a cited article, and the size reflects the number of co-citations, and the edges denote the co-cited relationships among articles.

Co-cited references are those articles cited together by other articles, and thus, can be regarded as the knowledge basis of a certain field. The knowledge map of the co-occurrence references reveals the developments and characteristics of this field (Figure 4). The nodes size, i.e., co-citations times, is generally larger than the previous one since 2007. The largest component of the co-citation network is divided into 41 clusters (size >1), which show the diversity of research topics. The top 10 articles by cited times and co-cited times are listed in Supplementary Tables 2, 3, respectively. A total of 2,115 articles are detected as citation bust, the highest strength is 185.33, and the longest duration is 9 years. Articles with high centrality are often considered as critical points or turning points in a field, and the top 10 articles are marked in Figure 4 and listed in Supplementary Table 4; their publication time range from 2002 to 2010.

### Present status of scientific collaboration and journal analysis

#### Country cooperation

**Figure 5:**
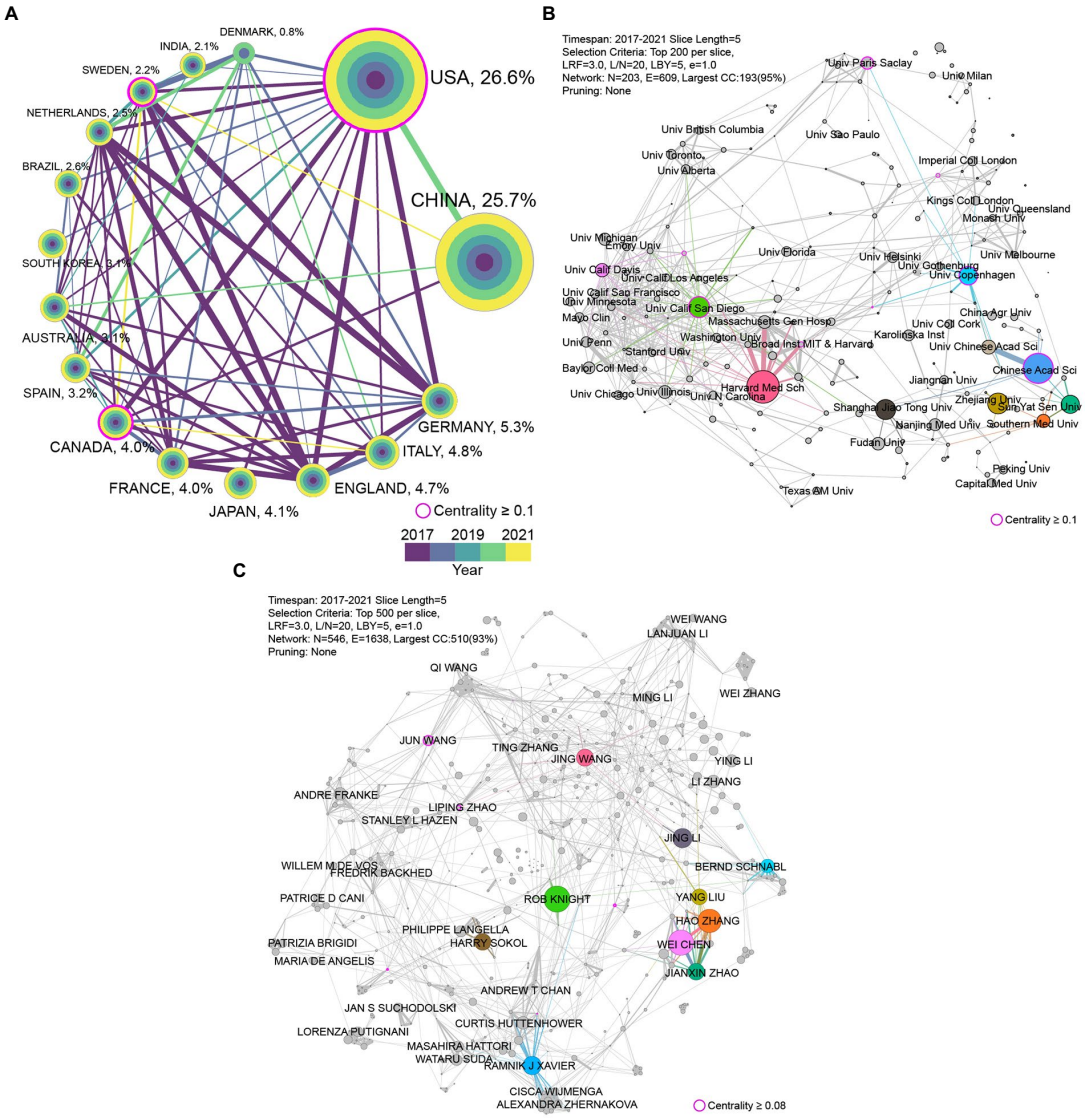
The cooperation network in different levels from 2017 to 2021. **(A)** The cooperation network of the top 15 most productive countries. The colored rings in the node represent publications amount in different years. The lines’ thickness and color indicate the strength of cooperation relationships and the year of first cooperation, respectively. **(B)** The largest component of cooperation network of institutions. The top 10 institutions in the number of publications are colored. **(C)** The largest component of author cooperation network. The top 10 authors in the number of publications are colored. The nodes’ size and the thickness of the lines positively correlated to the production of papers and the strength of cooperation relationships, respectively.

The data of publications in recent 5 years is utilized to evaluate the present cooperative status in the research filed of the gut microbiome and disease. A total of 18,049 articles were from 157 countries/regions in 2017–2021; the top 10 countries in terms of publications and centrality are shown in Supplementary Table 5. More than half of the publications were produced by the United States (*n* = 5,323) and China (*n* = 5,253), accounting for 29.5 and 29.1% of the total, respectively, while every other country contributed less than 6% of the total. Figure 5A shows the international research collaborations among the leading countries in papers output in this field. A higher centrality indicates that more information is passed through the node, which implies the importance of nodes in the network. The United States has the highest centrality value (0.52), followed by England (0.31) and Germany (0.14). Besides, the United States is the most active nation with the largest number of publications in this research filed. Although China’s publications amount is commensurate with the United States, it lagged behind in collaborations with other countries. Japan and India also had poor performance in collaborations among these top 15 countries.

#### Institution cooperation

There are 559 out of 12,186 institutions that participated in the publication of more than 25 articles from 2017 to 2021. The top 10 institutions in terms of publications and centrality are listed in Supplementary Table 6. Harvard Medical School had the largest number of publications (*n* = 329) among institutions worldwide, followed by the Chinese Academy of Sciences (*n* = 305) and the University of California San Diego (*n* = 225). The institutions cooperation network is shown in Figure 5B. The Harvard Medical School and the University of California San Diego are active institutions in this research filed in both publications and cooperation. There an obvious inner-country cooperation trend in the institutions cooperation network, especially in United States and China due to their large number of publications. But institutions cooperation network in the United States is more intensive than in China.

#### Author cooperation

Up to 79,972 authors were involved in the publication of the 18,049 articles from2017 to 2021, and a total of 218 authors participated in the publication of at least 25 articles. Detailed information on the top 10 authors in terms of publications and centrality is provided in Supplementary Table 7. Rob Knight is the most prolific author in the field of gut microbiome and disease, followed by Wei Chen and Hao Zhang. The author cooperation network is shown in Figure 5C. Similar to the institutions cooperation network, the inner-country cooperation pattern is also observed in the author cooperation network.

#### Journal analysis

114 out of 2,720 journals published more than 25 articles on the gut microbiome and disease over the 5 years. As shown in Supplementary Table 8, the top 20 journals with the highest number of articles included 4,356 records, which accounts for 24.13% of the total. Scientific reports are the most productive journal, with 605 articles in this field, followed by Frontiers in microbiology (510) and Plos one (409). Although Gut ranks 19th in terms of the number of articles published, it has the highest IF (23.059) among the 20 journals, followed by Nature communications (22.059) and Microbiome (14.650), and Gut is the most-cited journal with 92.879 citations per article. There was a significant positive correlation between impact factor values and the citations per article (R^2^ = 0.869, *p* < 0.001) for the top 20 most productive journals (Supplementary Figure 9).

### Hot topics and emerging trend

By combining Figure 2 with Figure 3, we can see that diseases that have attracted continuous attention are IBD, UC and CD. In contrast, other diseases have come into researchers’ notice in recent years, such as obesity, dysbiosis, diabetes, cardiovascular disease, Alzheimer’s disease, Parkinson’s disease, hypertension, depression and COVID-19. Compared to diet/nutrition and drugs, probiotics draw more attention, while FMT can be identified as a frontier of research.

**Figure 6:**
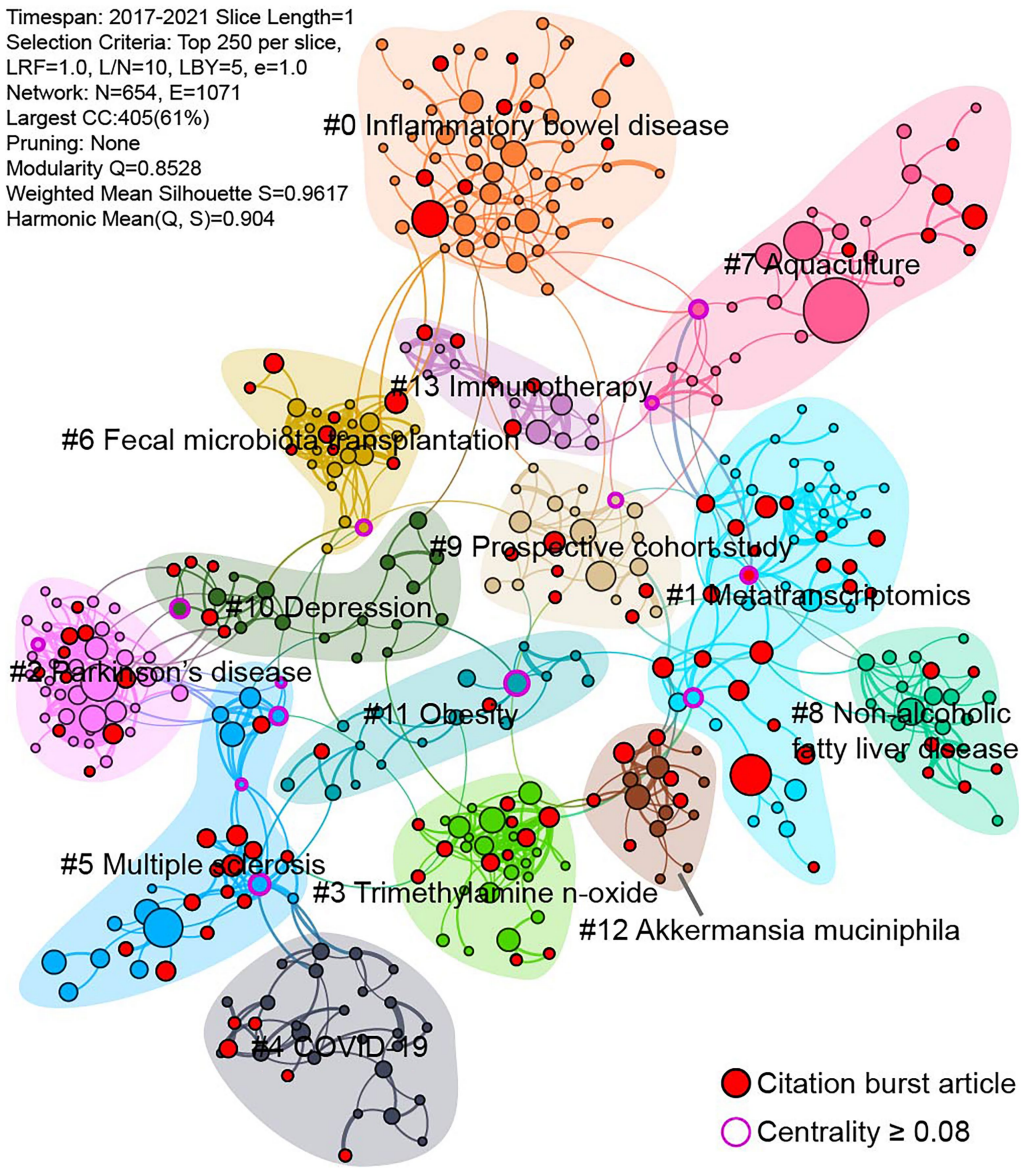
Co-cited references network from 2017 to 2021. Each node represents a cited article.

The data of publications in the recent 5 years is used to assess the current research status of gut microbiome and disease. The timeline view of keywords co-occurrence network reveals the development of gut microbiome and disease (Supplementary Figure 10). This network is divided into 21 clusters, which present the major subtopics in this field. Except “#0 growth performance” and “#14 oral microbiome” is irrelevant, other can be consider ongoing topics in recent. Campylobacter jejuni is commonly found in animal feces and causes human gastroenteritis, but the average year of “#16 campylobacter jejuni” is older than other clusters. In addition to utilizing keywords, co-cited references are also used to detect research hotspots and emerging trends. A total of 13 clusters are identified in the co-cited references network, and each cluster corresponds to a line of research (Figure 6). Except “#7 Aquaculture”, other clusters closely related to this field. Among these clusters, “#0 Inflammatory bowel disease” contains most of the nodes, which means that it has been widely reported. “#1 Metatrascriptomics” has most of the citation burst articles, followed by “#5 Multiple sclerosis,” “#3 trimethylamine N-oxide,” “#2 Parkinson’s disease,” which indicate they are active research areas.

### Diseases related to gut microbiome

**Figure 7:**
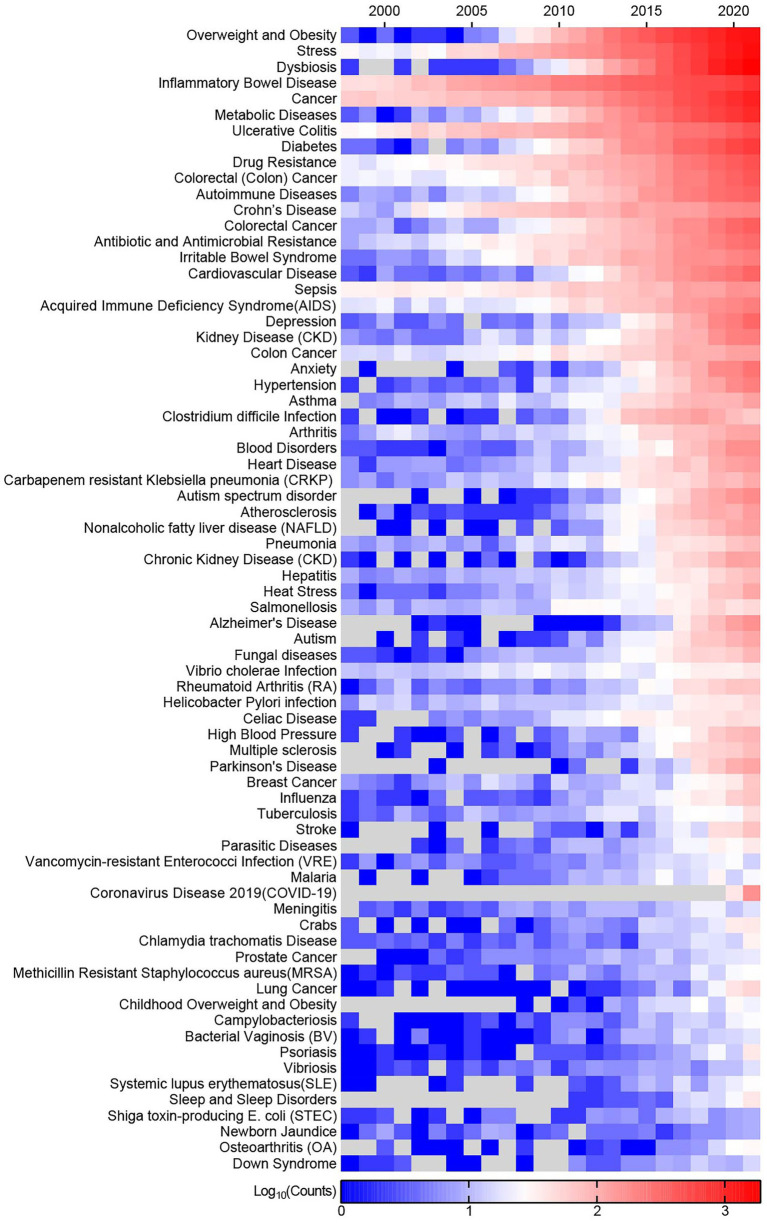
Publication trend of diseases and conditions related to gut microbiome from 1998 to 2021.

A total of 541 diseases and conditions are retrieved from the Centers for Disease Control and Prevention (CDC, https://www.cdc.gov/DiseasesConditions/) and the Illinois Department of Public Health (IDPH, https://dph.illinois.gov/topics-services/diseases-and-conditions.html), and they are used to search against WoSCC to depict research status of the gut microbiome with them. 73 diseases and conditions have more than 100 records in WoSCC from 1996 to 2021, and their publications trend are visualized in Figure 7. “Overweight and Obesity” is an area of focus, possessing the largest number of articles. While, “Stress” ranked second, possibly because of its lexical ambiguity and irrelevant articles are hit. Gut-related diseases have been more reported than others, which are consistent with previous results. There are 5,984 records related to gut microbiome and cancers, and 19 types of cancers are involved. Colorectal (Colon) Cancer is in the first echelon with the largest amount of records (*n* = 2,489), and the second echelon includes Breast Cancer, Prostate Cancer, Lung Cancer, Pancreatic Cancer, Leukemia and Liver Cancer, and others belongs to the third echelon with records less than 50 (Supplementary Figure 11).

## Discussion

Due to limitation of database, the earliest document is in 1996 in this study. But the first publication in this filed actually could trace back to 1958, when Eiseman et al. reported the successful treatment of pseudomembranous enterocolitis using a faecal enema (Eiseman et al., 1958). Studies about the gut microbiome and disease have increased tremendously over the last decades and present exponential growth, which revealed the important role of the gut microbiome in human health and disease (Gebrayel et al., 2022). Given that there are many unknowns about the gut microbiome and their potential applications in the prevention, diagnosis and therapy of diseases, this research scope will continue to attract keen interest among scientists, and further explorations will be conducted in the future.

To date, numerous studies have indicated that the intestinal microbiome is associated with various diseases, particularly digestive tract diseases (Nouvenne et al., 2018). However, most of them were observational (i.e., different in diversity, taxa, OTU and functions among groups) and did not reveal cause and effect (Koh and Bäckhed, 2020; Walter et al., 2020). It’s necessary to rethink whether there are causal relationships and whether the microbiota is a dominant or a crucial driving factor when surveying gut microbiome in diseases. Metabolites play an essential role in interactions between microbes and host cells, the altered composition of microbes could bring about a cascading impact on the immune system, and then effect the host health status (Rooks and Garrett, 2016). Currently, the most extensively studied metabolites are SCFA, bile acids, TMAO, and amino acid-derived metabolites (Liu et al., 2022), and other microbial metabolites such as lipids (Schoeler and Caesar, 2019), carbohydrates (Cheng et al., 2020) have also been proved to be essential for microbe-host interaction. However, comprehensive mechanisms that explain the link between the gut microbiome and most diseases remain poorly understood. Therefore, we encourage researchers to generate hypotheses based on observed differences in taxa and functions, and to independently validate it whenever possible.

The recent development of multi-omics approaches, such as metataxonomics [16S rRNA and ITS (Internal Transcribed Spacer) sequencing], shotgun metagenomics, metatranscriptomics, metaproteomics, and metabolomics, has enabled efficient characterization of microbial communities. These techniques not only provide the taxonomic profile of the microbial community but also assess their latent functions and metabolic activities (Zhang et al., 2019). The biomarkers detected by these -omics technologies could help to elucidate potential mechanisms of these commensals in health and disease (Lloyd-Price et al., 2019; Zhou et al., 2019; Mars et al., 2020). However, this also brings challenges to multi-omics data integration and mining (Whon et al., 2021). Currently, methods of data integration include two categories, i.e., multi-staged analysis and meta-analysis. Multi-staged integration means using two or more categorical features of the data. For example, metagenomics is combined with metabolomics (Oh et al., 2020). Meta-analysis attempts to systematically merge data across multiple studies and transform it into metadata that can be analyzed simultaneously (Armour et al., 2019; Wang et al., 2021; Drewes et al., 2022), which reduce study bias, increase statistical power and improve overall biological understanding of a study effect. As for data mining, machine learning has been applied to find biomarkers and carry out classification or prediction tasks, such as diagnosis, disease course, and disease severity (Marcos-Zambrano et al., 2021). But its limitation is requiring large amounts of data and lacking of interpretability. There are platforms and tools developed for multi-omics data integrating and mining, such as Qiita (Gonzalez et al., 2018), MicrobiomeAnalyst (Dhariwal et al., 2017), NetMoss (Xiao et al., 2022), tmap (Liao et al., 2019), which may aid in understanding the correlation between the gut microbiome and disease.

Besides investigating the relationship and mechanisms between the gut microbiome and diseases, it also is an interesting subject to modulate the gut microbiota to benefit health and reduce the risk of diseases. The main intervention strategies include diet/nutrition, dietary supplement, medicine and FMT. Diet is a feasible and easy measure to maintain homeostasis or increase the diversity of the gut microbiota. The question is, what type of diet can help to establish a good and stable intestinal microbiota (Leeming et al., 2019). Previous studies have indicated that FMT could restore gut microbial diversity and eliminate *Clostridioides difficile* infection (CDI; Kelly et al., 2021), which has encouraged research into the use of FMT for other diseases, such as ulcerative colitis and Crohn’s disease. While, results of FMT are not always desirable and the effectiveness is highly variable (Nie et al., 2019). It is assumed that the beneficial functions of therapeutic microbes are based on colonization and retention in sufficient quantity for enough time in recipients (Lee et al., 2017; Chu et al., 2021). Therefore, the selection of appropriate donors or its microbes and efficient colonization plays an essential role in patient response (Woodworth et al., 2017; Jouhten et al., 2020). It is also important to take into consideration how to appropriately evaluate the safety and efficacy for a given intervention (Green et al., 2020; Haifer et al., 2021). It is possible and valuable to develop novel diagnostic, prognostic and therapeutic strategies based on microbiome manipulation. The management of common diseases could be transformed by translating microbiome research into treatments that regulate the microbiome. Although there are some microbiome interventions as effective treatment for improving health conditions, its detail mechanisms are not fully understood.

As one of the hot topics in gut microbiome and diseases, IBD is a chronic inflammatory gut pathological condition, and represented by CD and UC. Both diseases are characterized by diarrhea, rectal bleeding, abdominal pain, fatigue and weight loss, but differentiate in clinical manifestations of inflammation and intestinal localization (Le Berre et al., 2020). Although a complete understanding of IBD pathogenesis is unclear, various risk factors associated with IBD have been identified, such as host genetic susceptibility, environmental variables, immune response and gut microbiome (Chang, 2020). Indeed, studies in human subjects have shown that the gut microbiome is significant different in patients with IBD compared with that in healthy individuals (Halfvarson et al., 2017; Lloyd-Price et al., 2019), such as reduced species richness and diversity, and lower temporal stability. Among them, the certain microbial taxa that are enriched or depleted in IBD, including bacteria, archaea, fungi, and viruses (Iliev and Cadwell, 2021), is usually interpreted as the imbalance between beneficial and pathogenic microbe, however, the results differ between studies (Schirmer et al., 2019). Alteration of gut microbial metabolites in IBD patients also detected, including fatty acids, amino acids and derivatives and bile acids, which may act as key regulators in the pathogenesis of IBD (Li et al., 2022; Paik et al., 2022). Although UC and CD are similar in epidemiologic, immunologic, therapeutic and clinical features, they fell into two distinct groups at the gut microbiome pattern (Pascal et al., 2017). The shifts in gut microbial community have been proven to be potential as diagnostic biomarkers of IBD (Zhou et al., 2018; Guo et al., 2022), which could be used to develop non-invasive diagnostic or monitor methods, while independent external validation is necessary before it can be used in clinic. There are therapeutic advances in gut microbiome modulation in patients with IBD, and a variety of microbiome-modulating interventions are proposed for treatment, such as probiotics, prebiotics, antibiotics, FMT, and dietary supplements (Eindor-Abarbanel et al., 2021). However, retrospective studies and meta-analyses on antibiotic use in UC and CD and long-term outcomes are controversial (Ledder, 2019). Similarly, the use of probiotics for the effective treatment of IBD remains inconclusive (Zhao et al., 2018). Due to the complexity and variety of IBD pathogenesis, personalized and multidimensional treatment will likely be required where microbiome-modulating therapy is coupled with other therapies. Changes in the gut microbiome seemed to play an important role in the onset of IBD, yet longitudinal studies of the gut microbiome are needed to move from association toward causation and modulation.

The research of the gut microbiome in human health and disease remains loaded with challenges. Gut microbiota is a complex and dynamic consortium influenced by multiple factors (Spencer et al., 2019; Kurilshikov et al., 2021; Gacesa et al., 2022). Changes in hosts’ lifestyle, such as diet, medication use, age, and socioeconomic status can lead to data reproducibility problems and statistical underpower. Recruiting participants with well-defined disease or at-risk conditions and well data management is important to reduce background noise. In addition, relatively few controlled samples in the trial may cause inconsistent results in the same disease. Because of the need for long longitudinal study, the influence from sample collection and storage and batch effects need to be avoided (Wang and LêCao, 2020; Poulsen Casper et al., 2021). Nowadays, gut microbiome research involves multi-disciplinary, not only microbiology and gastroenterology but also bioinformatics, mathematics, biochemistry, immunology and ecology, which pose challenges for single researcher (Mirzayi et al., 2021). There are gaps in scientific and technological power among countries, United States has established its leadership in this field. Therefore, we propose to enhance coordination and collaboration across the field among scientific communities to tackle shared challenges and explore new frontiers jointly. At present, inner-country cooperation pattern was observed at the institution and author levels, while a dynamic analysis of the collaboration networks based on different periods can show the evolution of collaborated patterns (Yu et al., 2020a). Effective international cooperation could promote academic exchanges. It may be a solution to the research of gut microbiome in disease by conducting well-designed large-scale cohort studies and randomized clinical trials, meanwhile combining multi-omics techniques and integrating microbiome data (Heintz-Buschart et al., 2016; Park et al., 2022). Due to confounding factors, it is necessary to establish standardized experimental procedures and subsequent data analysis pipelines (Szóstak et al., 2022). While experimental animal models can provide fascinating insights into the role of the microbiome in disease states, they rarely recapitulate the complete human phenotype (Hugenholtz and de Vos, 2018; Kieser et al., 2022). Therefore, extrapolations to human diseases have to be viewed with caution, and more rigorous experiments are required. The current focus concerning gut microbiota is mainly on bacteria, which neglects the significance of microbial intra- and inter-kingdom interaction. Fungi and viruses also impact the gut microbiota and host (van Tilburg Bernardes et al., 2020), although knowledge about their relationship with dysbiosis is limited (Carding et al., 2017; Beller and Matthijnssens, 2019). A recent study identified signature fungi in colorectal cancer and adenoma patients from multiple cohorts, and observed trans-kingdom interactions between enteric fungi and bacteria in colorectal cancer progression (Lin et al., 2022).

Bibliometric analysis is increasingly being used to assess hot topics and emerging areas of a specific field. Compared to narrative reviews that provide qualitative summary and commentary of published literature in a field, it quantitatively investigates the status of interdisciplinary fields based on citations and other statistical information regarding publications. In the future, the combination of the two will present a more precise historical context and future trajectory for a field. There are situations that need to be balanced in bibliometric analysis. The first situation is choosing databases. Other databases such as PubMed and Scopus also can be set as the data source, Scopus covers even more journals and also contains citation records. However, Web of Science (WoS) assigns document type labels more accurately than Scopus (Yeung, 2019), and we only filtered for original articles for the downstream analysis. The second situation is setting a search strategy. Well-defined search terms should include publications related to the field and exclude irrelevant ones as far as possible. It seems to be inevitable to contain irrelevant publications except for manual verification, but we believe that it is reliable to reflect the global trend and hot topics by these multi-aspect analysis. Artificial intelligence technology has the potential to realize semantic detection of publications and determine whether they belong to a specific theme. This would be especially useful for bibliometric analysis with massive volume of data and improve the accuracy of results.

Bibliometric methods are quantitative by nature to examine unlimited quantities of publications. But our study also comes with certain limitations. Firstly, due to the nature of the bibliometric methodology, the relationship between some bibliometric metrics and their assertions about research quality is often unclear (Wallin, 2005). Secondly, our study only retrieved data from WoS, yet a combination with other databases can be performed in similar type of research. Thirdly, synonymous words need be merged together during the analysis.

In conclusion, based on the detailed bibliometrics analysis of gut microbiome and disease, we present a comprehensive overview of this evolving subject over the past 26 years. These results indicate that gut microbiome and disease is an active research field, and publications on this subject have proliferated over the past decades. The current research mainly focuses on gastrointestinal diseases, while extra-intestinal diseases are also rising, such as nerve-related diseases. Although extensive correlative studies have been performed, the molecular mechanisms still need to be explored. Overall, gut microbiome research shows a multitude of challenges and great opportunities.

## Data availability statement

The original contributions presented in the study are included in the article/Supplementary material, further inquiries can be directed to the corresponding author.

## Author contributions

All authors contributed to the study conception and design. Material preparation, data collection and analysis were performed by ZH, KL, and WM. The first draft of the manuscript was written by ZH and all authors commented on previous versions of the manuscript. All authors contributed to the article and approved the submitted version.

## Funding

This project was supported by Science and Technology innovation Plan of Shanghai (19391902000).

## Conflict of interest

The authors declare that the research was conducted in the absence of any commercial or financial relationships that could be construed as a potential conflict of interest.

## Publisher’s note

All claims expressed in this article are solely those of the authors and do not necessarily represent those of their affiliated organizations, or those of the publisher, the editors and the reviewers. Any product that may be evaluated in this article, or claim that may be made by its manufacturer, is not guaranteed or endorsed by the publisher.

## Footnotes

^1^ https://clarivate.com/
