# Supplementary material for: The gut microbiome in human health and disease—Where are we and where are we going? A bibliometric analysis
Source: Front Microbiol. 2022 Dec 15;13:1018594. doi: 10.3389/fmicb.2022.1018594 (PMC9797740; doi:10.3389/fmicb.2022.1018594)
Supplement: Supplementary file 1 [file Data_Sheet_1.docx]

Supplementary Material

# Supplementary Figures and Tables

**Supplementary Table 1.** Types of documents published from 1996 to 2021.

| Document types^*^ | Count | Percentage (%) |
| --- | --- | --- |
| Articles | 29,870 | 66.074 |
| Review Articles | 13,311 | 29.445 |
| Proceedings papers | 1,065 | 2.356 |
| Editorial materials | 903 | 1.997 |
| Meeting abstracts | 592 | 1.310 |
| Book chapters | 369 | 0.816 |
| Early access | 228 | 0.504 |
| Letters | 140 | 0.310 |
| Corrections | 47 | 0.104 |
| News items | 23 | 0.051 |
| Data papers | 19 | 0.042 |
| Retracted publications | 11 | 0.024 |
| Reprints | 9 | 0.020 |
| Retractions | 5 | 0.011 |
| Biographical items | 3 | 0.007 |
| Software reviews | 2 | 0.004 |
| Notes | 1 | 0.002 |

^*^ There are overlaps in documents types, i.e., a publication can be included in one or more document types.

**Supplementary Table 2.** The top 10 articles by cited times in the field of human gut microbiome and disease.

| Rank | Title | Author | Year | Citation time | Journal |
| --- | --- | --- | --- | --- | --- |
| 1 | Metagenomic biomarker discovery and explanation | Segata Nicola, et al. | 2011 | 6,475 | GENOME BIOLOGY |
| 2 | Global, regional, and national prevalence of overweight and obesity in children and adults during 1980-2013: a systematic analysis for the Global Burden of Disease Study 2013 | Ng Marie, et al. | 2014 | 6,292 | LANCET |
| 3 | Diet rapidly and reproducibly alters the human gut microbiome | David Lawrence A., et al. | 2014 | 4,915 | NATURE |
| 4 | Diversity of the human intestinal microbial flora | Eckburg Paul B., et al | 2005 | 4,898 | SCIENCE |
| 5 | Metabolic endotoxemia initiates obesity and insulin resistance | Cani Patrice D., et al. | 2007 | 3,712 | DIABETES |
| 6 | A metagenome-wide association study of gut microbiota in type 2 diabetes | Qin Junjie, et al. | 2012 | 3,543 | NATURE |
| 7 | Impact of diet in shaping gut microbiota revealed by a comparative study in children from Europe and rural Africa | De Filippo Carlotta, et al. | 2010 | 3,274 | PROCEEDINGS OF THE NATIONAL ACADEMY OF SCIENCES OF THE UNITED STATES OF AMERICA |
| 8 | The Human Microbiome Project | Turnbaugh Peter J., et al. | 2007 | 3,108 | NATURE |
| 9 | Gut flora metabolism of phosphatidylcholine promotes cardiovascular disease | Wang Zeneng, et al. | 2011 | 3,055 | NATURE |
| 10 | Recognition of commensal microflora by toll-like receptors is required for intestinal homeostasis | Rakoff-Nahoum Seth, et al. | 2004 | 3,010 | CELL |

**Supplementary Table 3.** The top 10 articles by co-cited times in the field of human gut microbiome and disease.

| Rank | Title | Author | Year | Co-cited times | Journal |
| --- | --- | --- | --- | --- | --- |
| 1 | DADA2: High-resolution sample inference from Illumina amplicon data | Callahan Benjamin J., et al. | 2016 | 669 | NAT METHODS |
| 2 | Diet rapidly and reproducibly alters the human gut microbiome | David Lawrence A., et al. | 2014 | 570 | NATURE |
| 3 | The treatment-naive microbiome in new-onset Crohn's disease | Gevers Dirk, et al. | 2014 | 479 | CELL HOST MICROBE |
| 4 | Structure, function and diversity of the healthy human microbiome | Mitreva Makedonka, et al. | 2012 | 447 | NATURE |
| 5 | A human gut microbial gene catalogue established by metagenomic sequencing | Qin Junjie, et al. | 2010 | 432 | NATURE |
| 6 | Human gut microbiome viewed across age and geography | Yatsunenko Tanya et al. | 2012 | 418 | NATURE |
| 7 | Predictive functional profiling of microbial communities using 16S rRNA marker gene sequences | Langille Morgan GI, et al. | 2013 | 377 | NATURE BIOTECHNOLOGY |
| 8 | Reproducible, interactive, scalable and extensible microbiome data science using QIIME 2 | Bolyen Evan, et al. | 2019 | 375 | NATURE BIOTECHNOLOGY |
| 9 | Enterotypes of the human gut microbiome | Arumugam Manimozhiyan, et al. | 2011 | 363 | NATURE |
| 10 | From Dietary Fiber to Host Physiology: Short-Chain Fatty Acids as Key Bacterial Metabolites | Koh Ara, et al. | 2016 | 362 | CELL |

**Supplementary Table 4.** The top 10 co-cited references with highest centrality.

| Rank | Title | Author | Year | Centrality* | Co-cited times | Citation times |
| --- | --- | --- | --- | --- | --- | --- |
| 1 | Maintaining remission of ulcerative colitis with the probiotic Escherichia coli Nissle 1917 is as effective as with standard mesalazine | Kruis W., et al. | 2004 | 0.11 | 35 | 808 |
| 2 | Mucosal flora in inflammatory bowel disease | Swidsinski Alexander, et al. | 2002 | 0.09 | 129 | 1,066 |
| 3 | Oral bacteriotherapy as maintenance treatment in patients with chronic pouchitis: a double-blind, placebo-controlled trial | Gionchetti Paolo, et al. | 2000 | 0.09 | 82 | 1,106 |
| 4 | Alterations of the dominant faecal bacterial groups in patients with Crohn's disease of the colon | Seksik P., et al. | 2003 | 0.09 | 68 | 560 |
| 5 | Prophylaxis of pouchitis onset with probiotic therapy: a double-blind, placebo-controlled trial | Gionchetti Paolo, et al. | 2003 | 0.09 | 62 | 825 |
| 6 | Once daily high dose probiotic therapy (VSL#3) for maintaining remission in recurrent or refractory pouchitis | Mimura T., et al. | 2004 | 0.09 | 52 | 662 |
| 7 | Dysbiosis in inflammatory bowel disease | Tamboli Cyrus P., et al. | 2004 | 0.09 | 35 | 476 |
| 8 | Crohn's disease-associated adherent-invasive E.coli are selectively favoured by impaired autophagy to replicate intracellularly | Lapaquette Pierre, et al. | 2010 | 0.09 | 39 | 234 |
| 9 | Molecular Diversity of Escherichia Coli in the Human Gut: New Ecological Evidence Supporting the Role of Adherent-Invasive E. Coli (AIEC) in Crohn's disease | Martinez-Medina Margarita, et al. | 2009 | 0.09 | 16 | 263 |
| 10 | High prevalence of Escherichia coli belonging to the B2+D phylogenetic group in inflammatory bowel disease | Kotlowski, Roman, et al. | 2007 | 0.08 | 54 | 304 |

*The centrality of a node in a network measures the extent to which the node is part of paths that connect an arbitrary pair of nodes in the network. High values of centrality means the node are play an important role in the flow of information across the network, which indicate the importance of the node in scientific maps.

**Supplementary Table 5.** The top 10 countries in the number of publications and centrality from 2017 to 2021.

| Publications number | | | Centrality* | | |
| --- | --- | --- | --- | --- | --- |
| Ranking | Country | Value | Ranking | Country | Value |
| 1 | USA | 5,323 | 1 | USA | 0.52 |
| 2 | CHINA | 5,253 | 2 | ENGLAND | 0.31 |
| 3 | GERMANY | 1,048 | 3 | GERMANY | 0.14 |
| 4 | ITALY | 958 | 4 | JAPAN | 0.08 |
| 5 | ENGLAND | 929 | 5 | FRANCE | 0.08 |
| 6 | JAPAN | 830 | 6 | ITALY | 0.07 |
| 7 | CANADA | 806 | 7 | AUSTRALIA | 0.06 |
| 8 | FRANCE | 798 | 8 | NETHERLANDS | 0.06 |
| 9 | SPAIN | 650 | 9 | CANADA | 0.04 |
| 10 | AUSTRALIA | 626 | 10 | SPAIN | 0.03 |

*The centrality of a node in a network measures the extent to which the node is part of paths that connect an arbitrary pair of nodes in the network. High values of centrality means the node are play an important role in the flow of information across the network, which indicate the importance of the node in scientific maps.

**Supplementary Table 6.** The top 10 institutions in the number of publications and centrality from 2017 to 2021.

| Publications number | | | Centrality* | | |
| --- | --- | --- | --- | --- | --- |
| Ranking | Institution | Value | Ranking | Institution | Value |
| 1 | Harvard Med Sch | 329 | 1 | Univ Cambridge | 0.18 |
| 2 | Chinese Acad Sci | 305 | 2 | Univ Copenhagen | 0.17 |
| 3 | Univ Calif San Diego | 225 | 3 | Lund Univ | 0.14 |
| 4 | Zhejiang Univ | 224 | 4 | Univ Paris Saclay | 0.13 |
| 5 | Shanghai Jiao Tong Univ | 217 | 5 | Uppsala Univ | 0.13 |
| 6 | Univ Copenhagen | 202 | 6 | Univ Calif San Diego | 0.12 |
| 7 | Sun Yat Sen Univ | 200 | 7 | McGill Univ | 0.12 |
| 8 | Univ Chinese Acad Sci | 163 | 8 | Minist Educ | 0.12 |
| 9 | Univ Calif Davis | 158 | 9 | Broad Inst MIT & Harvard | 0.11 |
| 10 | Southern Med Univ | 156 | 10 | Chinese Acad Sci | 0.10 |

*The centrality of a node in a network measures the extent to which the node is part of paths that connect an arbitrary pair of nodes in the network. High values of centrality means the node are play an important role in the flow of information across the network, which indicate the importance of the node in scientific maps.

**Supplementary Table 7.** The top 10 authors in the number of publications and centrality from 2017 to 2021.

| Publications number | | | Centrality* | | |
| --- | --- | --- | --- | --- | --- |
| Ranking | Authors | Value | Ranking | Authors | Value |
| 1 | Rob Knight | 68 | 1 | B Brett Finlay | 0.10 |
| 2 | Wei Chen | 66 | 2 | Jun Wang | 0.09 |
| 3 | Hao Zhang | 62 | 3 | R Balfour Sartor | 0.09 |
| 4 | Jing Li | 53 | 4 | Danilo Ercolini | 0.09 |
| 5 | Ramnik J Xavier | 51 | 5 | Liping Zhao | 0.08 |
| 6 | Jing Wang | 48 | 6 | Clary B Clish | 0.08 |
| 7 | Jianxin Zhao | 47 | 7 | Tao Zhang | 0.08 |
| 8 | Harry Sokol | 47 | 8 | Liang Chen | 0.07 |
| 9 | Yang Liu | 45 | 9 | Eric A Franzosa | 0.07 |
| 10 | Bernd Schnabl | 40 | 10 | Yoshiki Vazquezbaeza | 0.07 |

*The centrality of a node in a network measures the extent to which the node is part of paths that connect an arbitrary pair of nodes in the network. High values of centrality means the node are play an important role in the flow of information across the network, which indicate the importance of the node in scientific maps.

**Supplementary Table 8.** List of the top 20 journals publishing research on gut microbiome and disease from 2017 to 2021.

| Journal | Publications | Percentage (%) | JCR^TM^ partition | IF_2021_ | Times Cited | Average Times Cited |
| --- | --- | --- | --- | --- | --- | --- |
| Scientific reports | 605 | 3.351 | Q1 | 4.379 | 14,293 | 23.625 |
| Frontiers in microbiology | 510 | 2.825 | Q1 | 5.640 | 9,697 | 19.014 |
| Plos one | 409 | 2.266 | Q2 | 3.240 | 6,788 | 16.597 |
| Nutrients | 308 | 1.706 | Q1 | 5.717 | 4,401 | 14.289 |
| Frontiers in immunology | 285 | 1.579 | Q1 | 7.561 | 4,548 | 15.958 |
| Food function | 225 | 1.246 | Q1 | 5.396 | 2,524 | 11.218 |
| Gut microbes | 197 | 1.091 | Q1 | 10.245 | 3,212 | 16.305 |
| Frontiers in cellular and infection microbiology | 185 | 1.025 | Q1 | 5.293 | 2,382 | 12.876 |
| Microbiome | 168 | 0.931 | Q1 | 14.65 | 7,226 | 43.012 |
| Microorganisms | 164 | 0.908 | Q2 | 4.128 | 928 | 5.659 |
| Aquaculture | 159 | 0.881 | Q1 | 4.242 | 2,148 | 13.509 |
| Journal of functional foods | 139 | 0.77 | Q1 | 4.451 | 1,463 | 10.525 |
| International journal of molecular sciences | 136 | 0.753 | Q1 | 5.923 | 1,191 | 8.757 |
| Fish shellfish immunology | 133 | 0.737 | Q1 | 4.581 | 2,836 | 21.323 |
| Nature communications | 132 | 0.731 | Q1 | 14.919 | 7,139 | 54.083 |
| Journal of agricultural and food chemistry | 128 | 0.709 | Q1 | 5.279 | 1,981 | 15.477 |
| mSystems | 128 | 0.709 | Q1 | 6.496 | 2,433 | 19.008 |
| Frontiers in pharmacology | 118 | 0.654 | Q1 | 5.810 | 993 | 8.415 |
| Gut | 116 | 0.643 | Q1 | 23.059 | 10,774 | 92.879 |
| Animals | 111 | 0.615 | Q1 | 2.752 | 550 | 4.9545 |

JCR^TM^: journal of citation reports, IF_2021_: impact factor in 2021


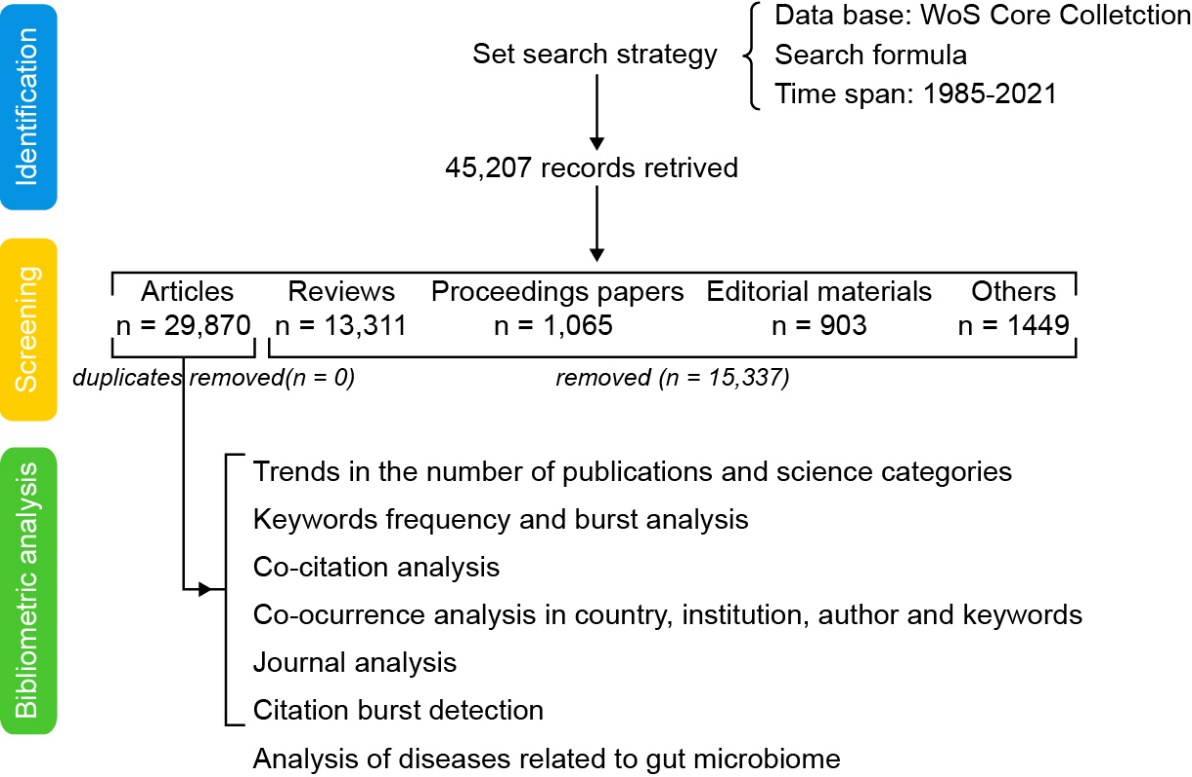


**Supplementary Figure 1.** Flowchart of the bibliometric analysis in this study.


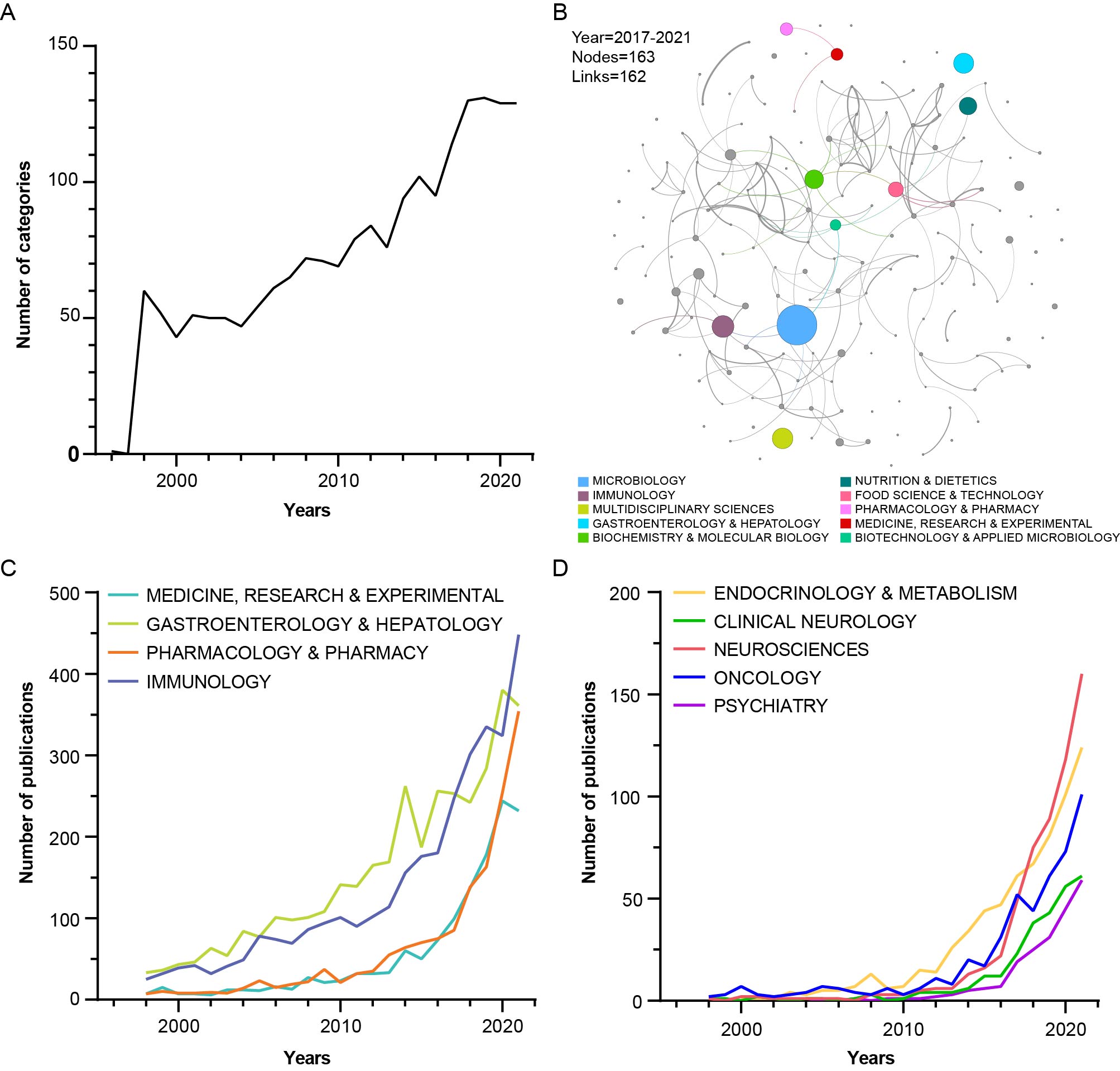


**Supplementary Figure 2.** The changing trend in science categories. **(A)** The number of subject categories each year. **(B)** The co-occurrence network of science categories in 2017-2021. The top 10 categories by frequency are colored. **(C)** The number of articles published in the top 4 science categories related to medicine each year. **(D)** The Number of articles published in the top 5 science categories related to nerve each year.


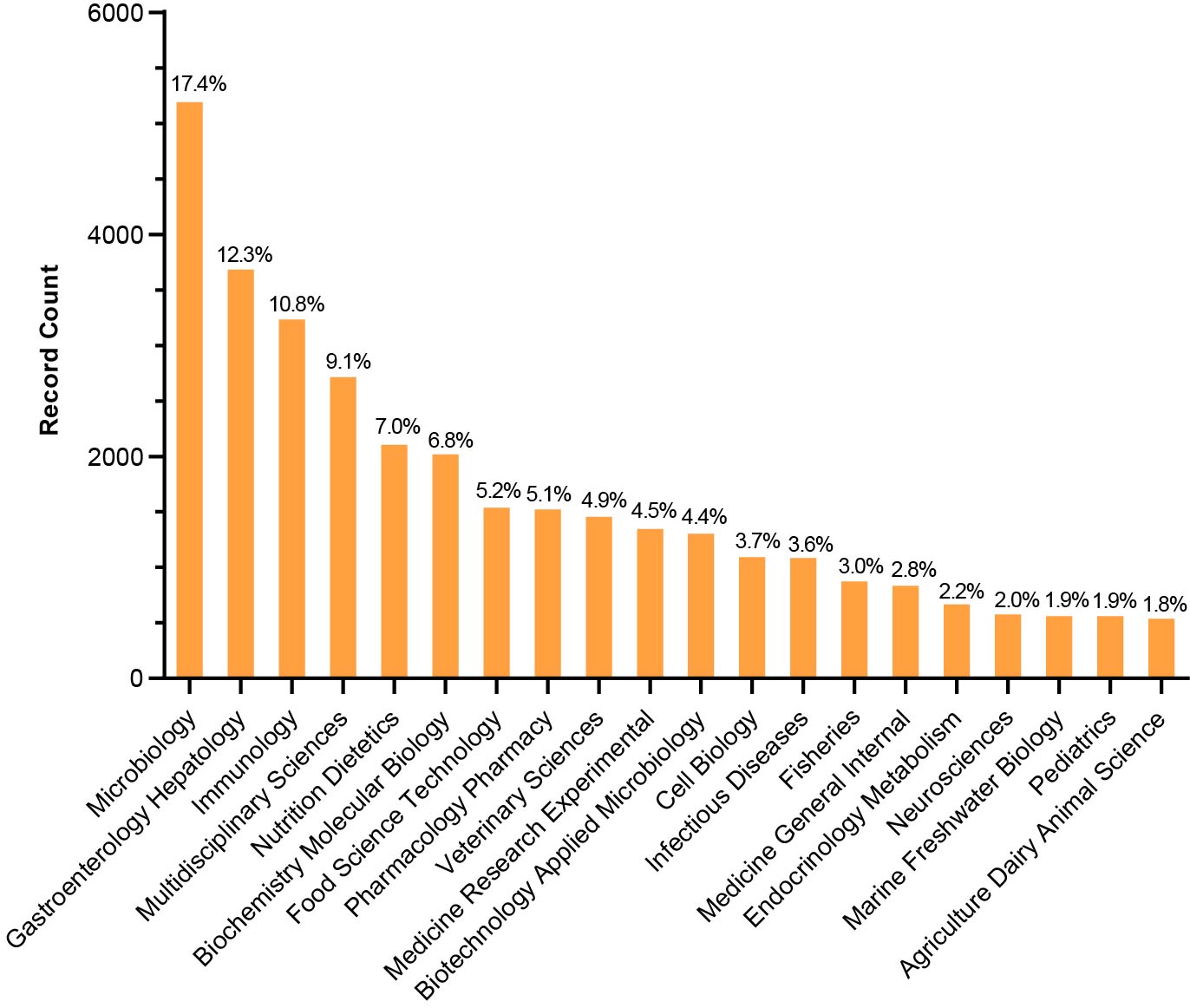


**Supplementary Figure 3.** Distribution of the 29,870 articles in Web of Science Categories (top 20) in 1996–2021. An article can be included in one or more categories. Percentage = record count/number of all articles.


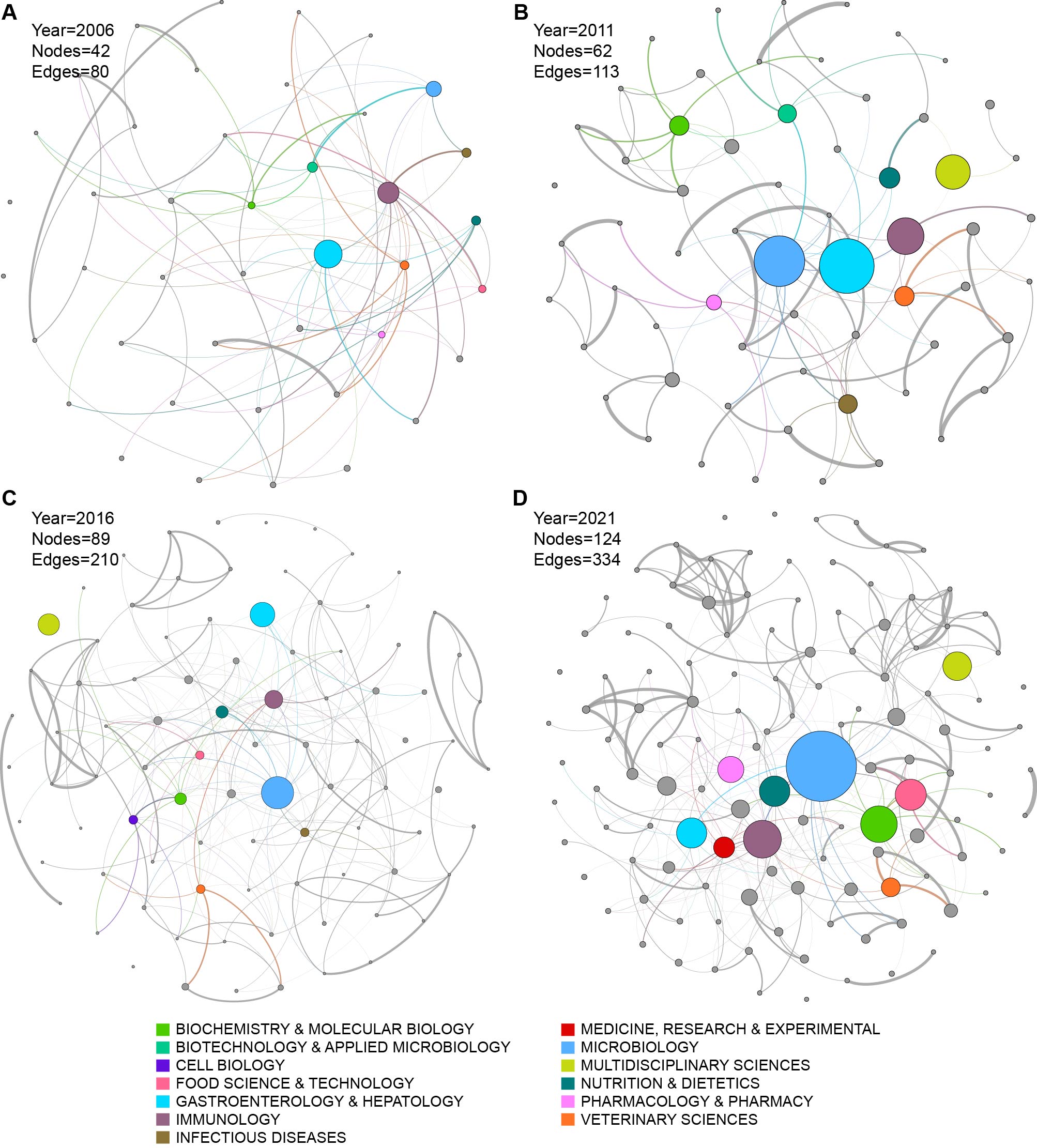


**Supplementary Figure 4.** Co-occurrence network of science categories different years. **(A)** In 2006. **(B)** In 2011. **(C)** In 2016. **(D)** In 2021.


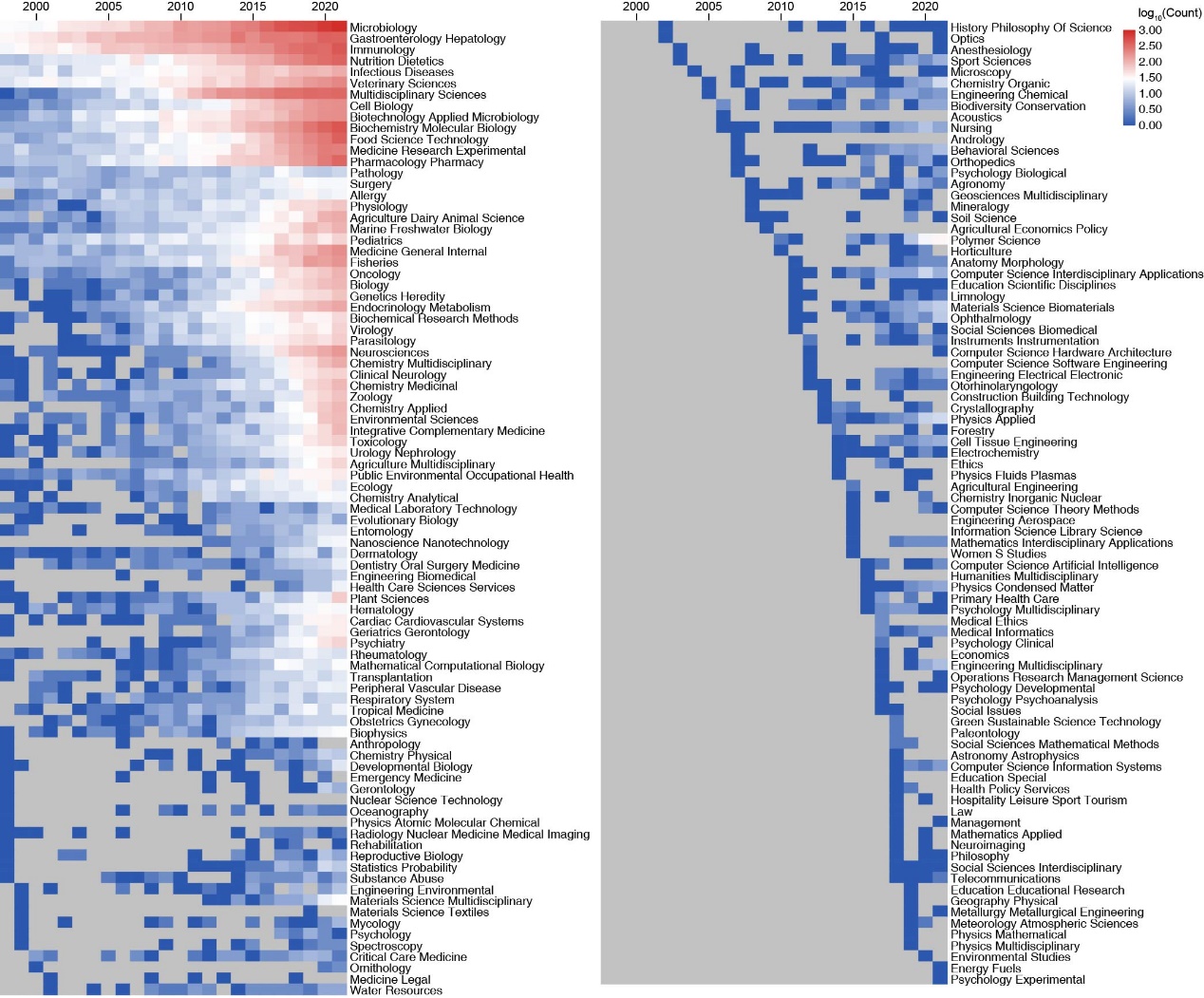


**Supplementary Figure 5.** Heatmap of subject categories of the publication in 1998-2021.


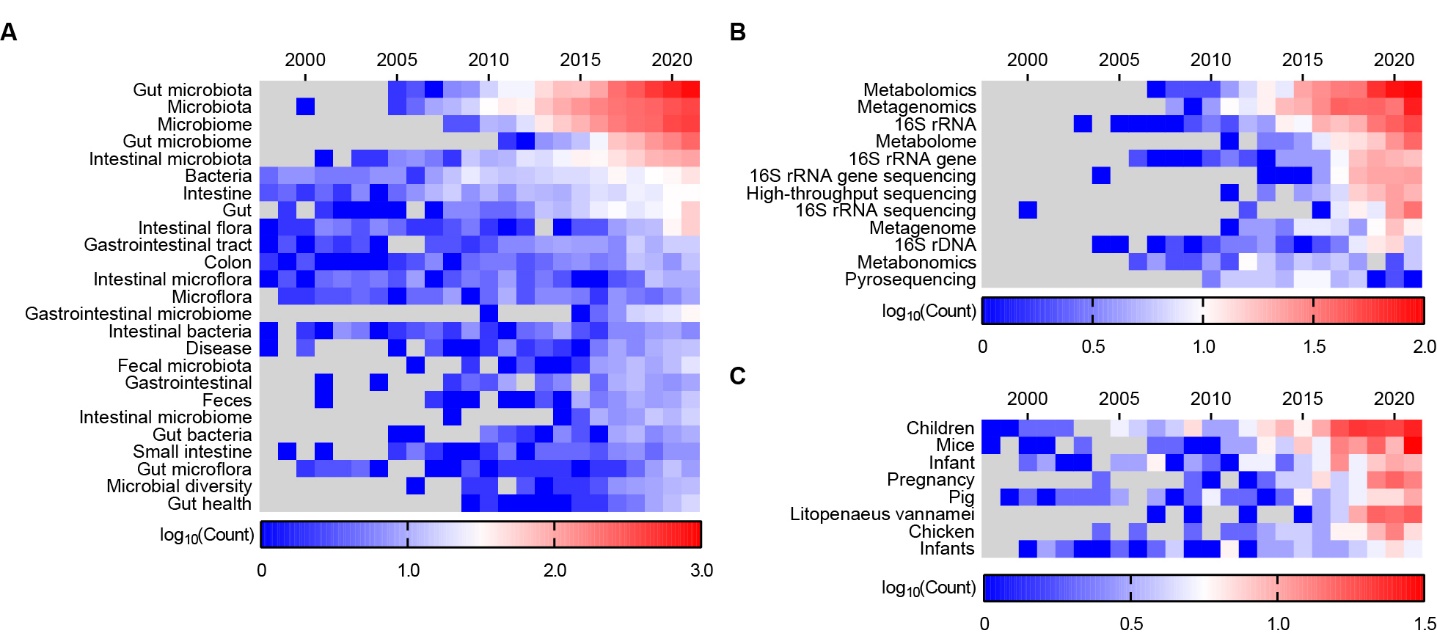


**Supplementary Figure 6.** Heatmap of keywords of the articles published in 1998-2021. **(A)** Keywords related to definition of the field. **(B)** Keywords related to technology. **(C)** Keywords related to research subject.


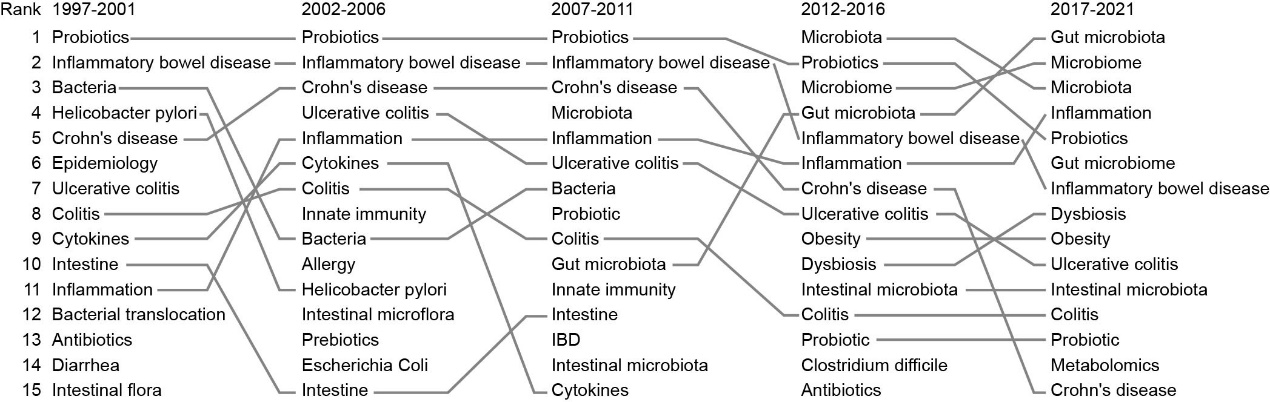


**Supplementary Figure 7.** Top 15 keywords by frequency in every five years slice from 1997 to 2021.


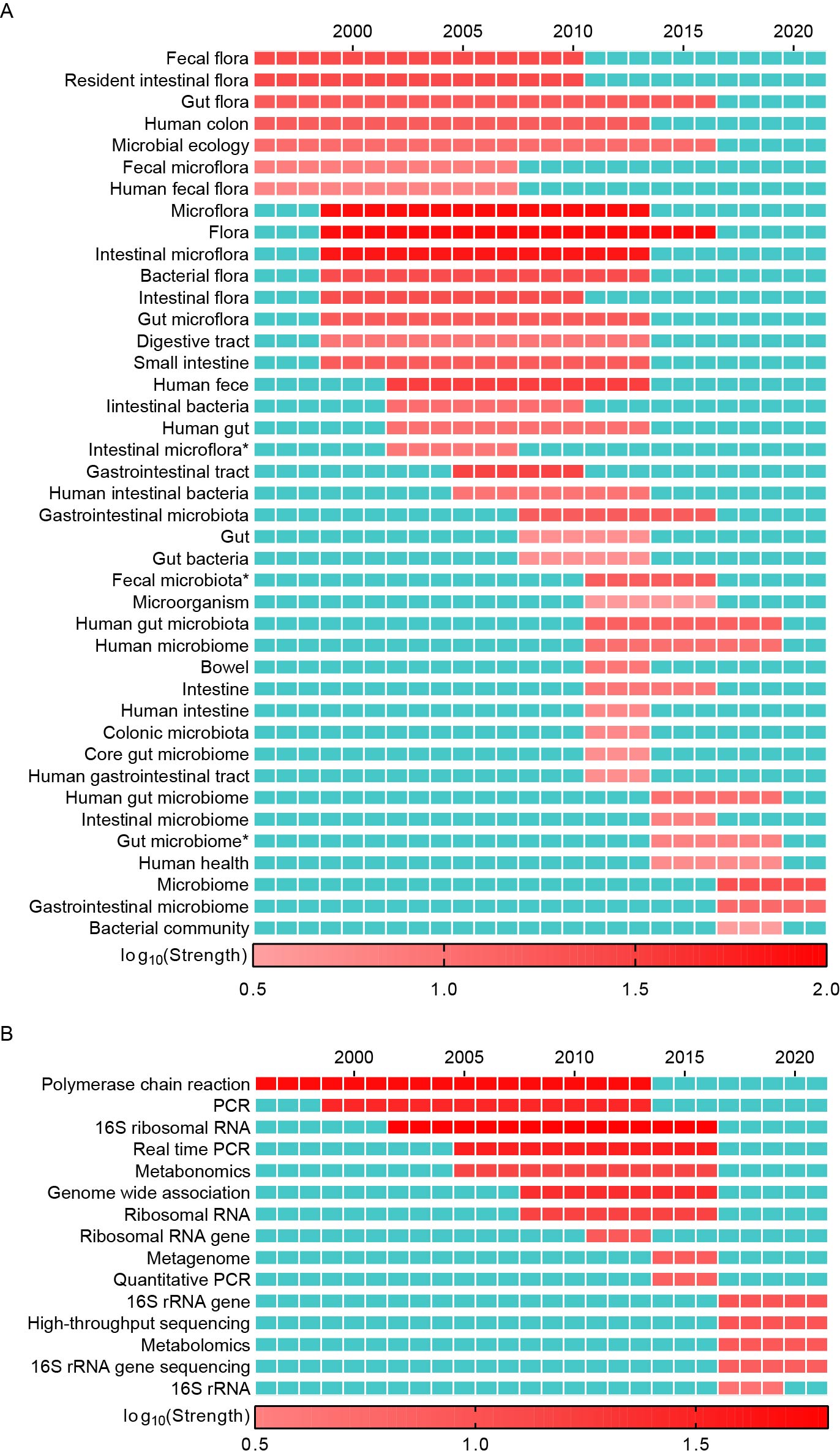


**Supplementary Figure 8.** Keywords burst visualization. **(A)** Bursting keywords that define the research area. **(B)** Technological keywords burst in 1996-2021. * indicate the origin keywords missed blank and were corrected. Bar in red means burst event is detected in that year and color density indicate the burst strength, while bar in green means no burst event in that year.


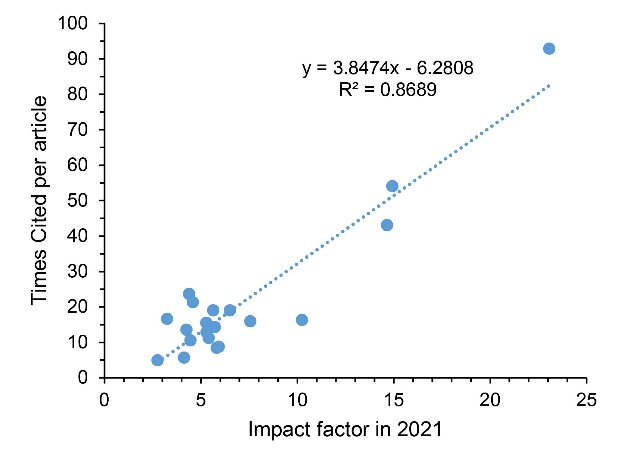


**Supplementary Figure 9.** Simple linear regression analysis representing the association of times cited per article with impact factors.


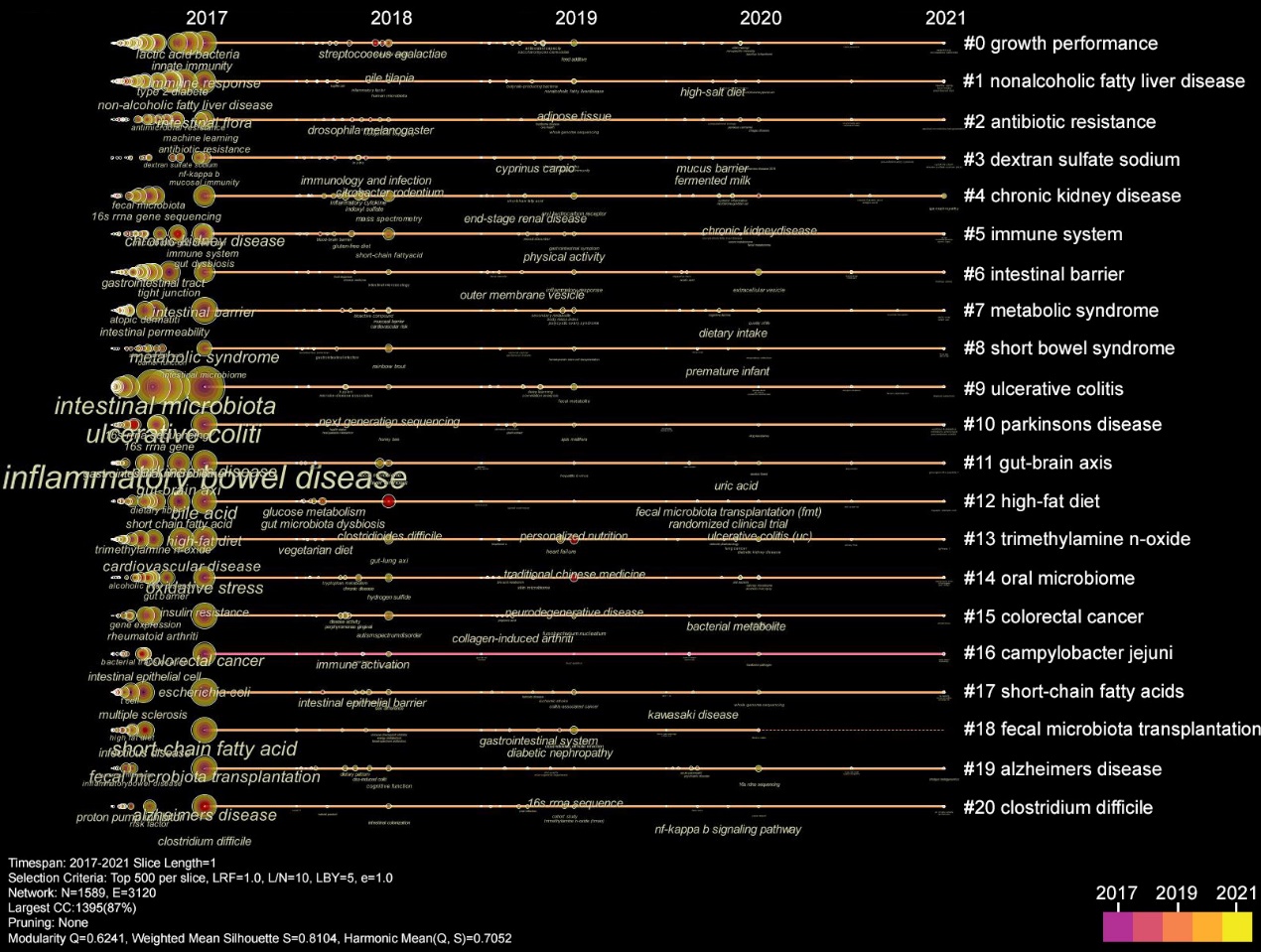


**Supplementary Figure 10.** The co-occurrence network of keywords in timeline view. Clusters are arranged on the right of the timeline. The color of a timeline presents the average year of the corresponding cluster.


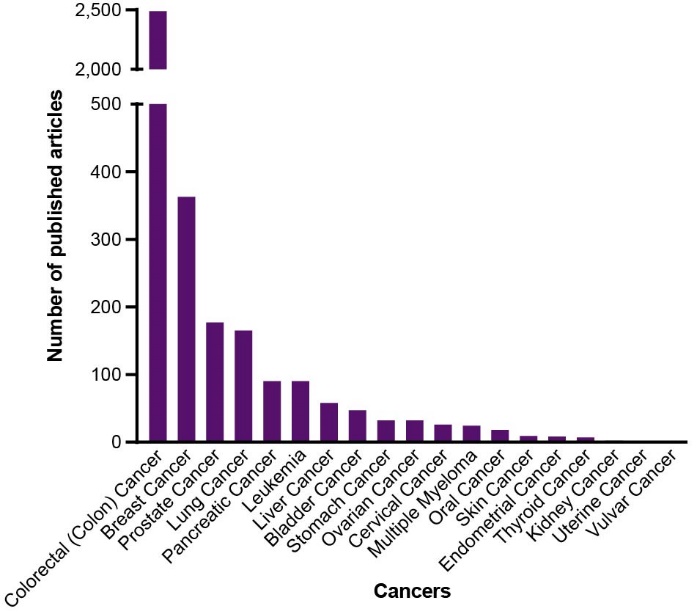


**Supplementary Figure 11.** Article records of cancers related to gut microbiome from 1993 to 2021 in WoSCC.
